# Supplementary material for: Comparing Computational Peritoneal Dialysis Models in Pigs and Patients
Source: Toxins (Basel). 2025 Jun 28;17(7):329. doi: 10.3390/toxins17070329 (PMC12298135; doi:10.3390/toxins17070329)
Supplement: Supplementary file 1 [file toxins-17-00329-s001.zip › toxins-3698358-supplementary.pdf]

Supplementary material

CONTENTS

1. Model descriptions 2
1.1. Unified Graff model 2
1.2. Three-pore model 3
1.3. Garred model 5
1.4. Waniewski model 5
2. Population average of fitted parameters across 23 pigs and 20 human 6
3. Subject-specific simulations 8
3.1. Specific-pig simulations 8
3.1.1. Predicted values for 6 models in a representative pig dwell (1) 8
3.1.2. Predicted parameter values for 6 models in representative pig dwell (2) 9
3.1.3. Predicted parameter values for 6 models in representative pig session (3) 10
3.1.4. Predicted parameter values for 6 models in representative pig dwell (4) 11
3.2. Specific-human simulation 12
3.2.1. Predicted parameter values for 6 models in representative human dwell (1) 12
3.2.2. Predicted parameter values for 6 models in representative human dwell (2) 13
3.2.3. Predicted parameter values for 6 models in representative human dwell (3) 13
3.2.4. Predicted parameter values for 6 models in representative human dwell (4) 14
3.3. Population-average of fitted parameters 15
3.3.1. Fitted and derived fct values 15
3.3.2. Fitted and derived sieving coefficient values 16
3.4. Correlation matrix 17
3.5. Avenues for personalisation of treatment for patients 18
References 19

1. Model descriptions

1.1. Unified Graff model

Graff and Fugleberg *et al.*, in a series of articles compared 6 models of transport for the peritoneal solute transport of urea, creatinine, glucose, sodium, potassium and phosphate[1-6]. The solute flux is given by a combination of three main processes: diffusion, convection from blood to dialysate and lymphatic absorption from the dialysate. It is assumed that there is no solute generation in the peritoneal tissue or the peritoneal cavity (the time frame is too small) and the interactions between the solutes are negligible.

The solute flux is given by,

$$\frac{dc_D}{dt} = \frac{1}{\underbrace{V}_{ml}} * \left(\underbrace{\overbrace{MTAC}^{ml/min} * \left(\overbrace{fct}^{-} * \overbrace{c_p}^{mol/ml} - c_D \right)}_{diffusive} + \underbrace{\overbrace{SiCo}^{-} * \overbrace{UF}^{ml/min} * \overbrace{c_I}^{mol/ml}}_{Convective} - \underbrace{\overbrace{L}^{ml/min} * \overbrace{c^*}^{\frac{mol}{ml}}}_{lymphatic} - c_D * UF \right) \quad 1.1$$

where,

c_D is the dialysate concentration at time t,

V is the peritoneal volume at time t,

$MTAC$ is the mass transfer area coefficient of the particular solute (fitted),

fct is the equilibrium ratio for solute concentration in dialysate and plasma concentration $\left(\frac{c_D}{c_p}\right)$ (fitted),

c_p is the plasma concentration at time t,

$SiCo$ is the solute specific sieving coefficient to account for the fraction of molecules dragged along during the water transport (fitted),

UF is the ultrafiltration rate given by $\frac{V(t+\Delta t)-V(t)}{\Delta t} + L$,

L is the lymphatic flow rate (0.3 ml/min for pigs and 0.3 ml/min for human). The lymphatic flow is modelled as a sum total of different water transport mechanisms such as the dialysate sampling, lymphatic entry and blood entry,

c_I is the intramembrane solute concentration to account for the non-lymphatic convective transfer across the peritoneal membrane given by $c_p - f(c_p - c_d)$,

f is the interdependence between diffusion and convection given by,

$$f = \frac{1}{\beta} - \frac{1}{\beta - 1}$$

and $\beta = UF * SiCo/MTAC$.

c^* is to account for the direction of the lymphatic flow ($c^* = c_p$ if flow is from plasma to peritoneal cavity or else $c^* = c_D$).

For the UGM, we combine the best fits predicted by Graff and Fugleberg et al. (parameters in **Table S1**) to predict the dialysate solute concentration of the six solutes simultaneously. We also fit all parameters to check if that improves the fit (UGM-18).

Table S1: Different mechanisms proposed for various solute transfer during dwells.

Solute	$\boldsymbol{MTAC}$ ml/min	$\boldsymbol{fct}$	$\boldsymbol{SiCo}$	$\boldsymbol{L}$ ml/min
Urea	fitted	1	fitted	
Sodium	fitted	0.94	fitted	
Phosphate	fitted	1	fitted	0.3 (pigs)
Potassium	fitted	0.96	fitted	0.3 (human)
Creatinine	fitted	1	fitted	
Glucose	fitted	1	0	

1.2. Three-pore model

Rippe's TPM describes fluid transport across the endothelial barrier[7]. It proposes three pathways: 1. A large-pore pathway allowing for rapid macromolecule exchange, 2. A small-pore pathway permitting slower transport of small solutes, and 3. An ultra-small-pore pathway for the movement of water.

$$\frac{dV}{dt} = J_{vC} + J_{vS} + J_{vL} - L \quad 1.2$$

The volume flux is given by,

and the solute flux is given by

$$\frac{dC_D}{dt} = \underbrace{\frac{J_{sC} + J_{sS} + J_{sL}}{V}}_{Solute\ flux} - \underbrace{C_D \frac{J_{vC} + J_{vS} + J_{vL}}{V}}_{Dilution/Concentration} \qquad 1.3$$

The second term in equation 1.3 refers to the dilution or concentration of the solute in the abdominal cavity in response to the ultrafiltration.

where

$$J_{vC} = L_pS * [\Delta P - \Delta\pi] * af * \alpha_C \qquad 1.4$$

$$J_{vS} = L_pS * [\Delta P - \sigma_S\Delta\pi] * af * \alpha_S$$

$$J_{vL} = L_pS * [\Delta P - \sigma_L\Delta\pi] * af * \alpha_L$$

and

$$\Delta P = \Delta P_0 - \frac{V[t] - (V_{fill} + V_{res})}{490} \qquad 1.5$$

Where J_{vC},J_{vS} and J_{vL} are the volume flux through ultrasmall, small and large pores, LpS is the effective hydraulic conductivity (0.074 ml/min mmHg), af is the fraction of peritoneum in contact with the dialysis fluid ($af = 16.18 * (1 - np.exp\ (-0.00077 * V[t]))/13.3187$) and α_C, α_S and α_L are the ultrasmall, small and large pore fraction. The fill volume and residual volume are represented by V_{fill} and V_{res}. The solute flux through the small and large pores is given by,

$$J_{sS} = J_{vS}(1 - \sigma_S)\frac{c_p - c_D e^{-Pe,S}}{1 - e^{-Pe,S}} \qquad 1.6$$

$$J_{sL} = J_{vL}(1 - \sigma_L)\frac{c_p - c_D e^{-Pe,L}}{1 - e^{-Pe,L}}$$

There is no solute flow through the ultrasmall pores (water-exclusive pores).

$$J_{sC} = 0. \qquad 1.7$$

where

$$Pe, S = \frac{J_{vS}(1 - \sigma_S)}{MTAC} \qquad 1.8$$

$$Pe, L = \frac{J_{vL}(1 - \sigma_L)}{MTAC}$$

The reflection coefficients for solutes are given by σ[8] ($\sigma = 1 - SiCo$).

1.3. Garred model

The simplified model proposed by Garred *et al.*[9] is given by,

$$\ln\{V(\bar{c_p} - c_D)\} = \ln\{V[0](\bar{c_p} - c_D[0])\} - \frac{MTAC}{\bar{V}} \times t \tag{1.9}$$

where V is the dialysate volume, $\bar{c_p}$ is the mean plasma concentration during the session, c_D is the dialysate concentration at time t, $\bar{V}$ is the mean dialysate volume during the session, $V[0]$ and $c_D[0]$ are the dialysate volume and dialysate concentration at time 0. They assume that there is no interdependence of the two transport processes- diffusion and convection, which is fixed to zero ($f = 0$). Further they also assumed that for small solutes, the sieving coefficient or $SiCo$ can be set to 1. Plotting a straight line through $\ln\{V(\bar{c_p} - c_D)\}$ for two different time points can then give us the slope $\frac{MTAC}{\bar{V}}$ from which $MTAC$ can then be derived. Using this predicted $MTAC$, we can calculate c_D as,

$$c_D = \bar{c_p} - \frac{V[0]}{V}(\bar{c_p} - c_D[0]) \exp\left(-\frac{MTAC}{\bar{V}} \times t\right) \tag{1.10}$$

1.4. Waniewski model

The mass balance of the solute in the dialysate is given by,

$$\frac{dV_D c_D}{dt} = Q_s$$

$$Q_s = MTAC * (c_p - c_D) + SiCo * UF * \bar{\bar{c}}$$

$$\bar{\bar{c}} = (1 - f)c_p + f c_D$$

where UF and f is calculated in the same way as above.

The WM simplifies the model by the assumption, f = constant.

$$V[t] * c_D[t] = V[0] * c_D[0] + MTAC * X_1[t] + SiCo * X_2[t] \tag{1.11}$$

where

$$X_1[t] = \int_0^t (c_p - c_D)dt$$

$$X_2 = \int_0^t UF((1 - f)c_p + f c_D)dt$$

Waniewski also modified the simplistic model of Garred by making one change, i.e., instead of assuming $f = 0$ for all solutes, they assumed f is a constant[10]. Thus in the simple Waniewski model (SWM), equation 1.9 can be modified as

$$\ln\{V^{1-f}(\bar{c_p} - c_D)\} = \ln\{(V[0])^{1-f}(\bar{c_p} - c_D[0])\} - \frac{MTAC}{\bar{V}} \times t \qquad 1.12$$

and this gives us the dialysate concentration as,

$$c_D = \bar{c_p} - \left(\frac{V[0]}{V}\right)^{1-f} (\bar{c_p} - c_D[0])\exp\left(-\frac{MTAC}{\bar{V}} \times t\right) \qquad 1.13$$

2. Population average of fitted parameters across 23 pigs and 20 human

Table S2: Population average (mean ± SD) predicted model parameters in pigs and patients. Human parameters are shown in bold. Data for six solutes (Urea, Creatinine, Sodium, Phosphate, Glucose, Potassium) were collected and fitted in pigs while data for a subset of solutes (Urea, Creatinine and Glucose) were collected and fitted in human. MTAC = mass transfer area coefficient (ml/min). fct = relationship between diffusion and convection, particular only to Graff models (dimensionless). SiCo = Sieving coefficient (dimensionless). L = Lymphatic flow rate (ml/min), Weight (kg).

		TPM	GM	WM	SWM	UGM	UGM-18
**$MTAC$ (ml/min)**	Urea	10.76 ± 4.59	10.27 ± 4.50	11.55 ± 7.23	9.89 ± 3.32	10.69 ± 4.30	9.14 ± 4.81
		**24.82 ± 11.49**	**20.48 ± 5.90**	**15.46 ± 10.45**	**21.54 ± 6.11**	**24.33 ± 8.80**	**24.52 ± 9.16**
	Creatinine	5.00 ± 2.59	5.58 ± 4.10	7.40 ± 6.67	5.21 ± 2.75	5.42 ± 2.61	4.22 ± 4.74
		**17.62 ± 7.35**	**13.21 ± 5.42**	**12.03 ± 6.34**	**14.27 ± 5.47**	**15.20 ± 6.53**	**15.23 ± 6.57**
	Sodium	1.00 ± 1.27	-1.15 ± 4.55	-24.96 ± 203.19	-1.52 ± 3.20	3.33 ± 1.78	2.01 ± 2.14
		**13.29 ± 11.67**	**-11.14 ± 9.63**	**4.19 ± 93.98**	**-10.84 ± 10.08**	**65.09 ± 87.40**	**26.79 ± 65.19**
	Phosphate	3.85 ± 2.05	4.52 ± 3.89	6.32 ± 4.44	4.14 ± 2.44	4.35 ± 2.43	3.72 ± 2.79
	Glucose	5.64 ± 2.35	6.54 ± 3.93	21.92 ± 61.38	6.16 ± 2.63	6.56 ± 5.03	5.73 ± 3.12
		**22.66 ± 10.16**	**16.61 ± 6.00**	**51.06 ± 37.59**	**17.67 ± 6.16**	**23.53 ± 11.69**	**25.00 ± 11.64**
	Potassium	20.30 ± 2.93	18.16 ± 4.85	40.02 ± 12.34	17.79 ± 4.14	24.46 ± 7.39	21.31 ± 7.53
**fct**	Urea		0[a]	0[a]	0.5[a]	1[a]	0.91 ± 0.29
			**0[a]**	**0[a]**	**0.5[a]**	**1[a]**	**1.00 ± 0.00**
	Creatinine		0[a]	0[a]	0.5[a]	1[a]	0.65 ± 0.49
			**0[a]**	**0[a]**	**0.5[a]**	**1[a]**	**1.00 ± 0.00**

	Sodium		0[a]	0[a]	0.5[a]	0.94[a]	0.61 ± 0.50
			**0[a]**	**0[a]**	**0.5[a]**	**0.94[a]**	**1.00 ± 0.00**
	Phosphate		0[a]	0[a]	0.5[a]	1[a]	0.78 ± 0.42
	Glucose		0[a]	0[a]	0.5[a]	1[a]	0.57 ± 0.51
			**0[a]**	**0[a]**	**0.5[a]**	**1[a]**	**1.00 ± 0.00**
	Potassium		0[a]	0[a]	0.5[a]	0.96	0.96 ± 0.21
SiCo	Urea	0.963 [a]		0.04 ± 0.04		0.57 ± 0.51	0.52 ± 0.51
		**0.963 [a]**		**-0.01 ± 0.05**		**1.00 ± 0.00**	**1.00 ± 0.00**
	Creatinine	0.958 [a]		0.05 ± 0.08		0.61 ± 0.50	0.43 ± 0.51
		**0.958 [a]**		**-0.02 ± 0.04**		**1.00 ± 0.00**	**1.00 ± 0.00**
	Sodium	0.967 [a]		-0.02 ± 0.35		0.96 ± 0.21	0.52 ± 0.51
		**0.967[a]**		**-0.04 ± 0.11**		**1.00 ± 0.00**	**1.00 ± 0.00**
	Phosphate	0.961 [a]		0.06 ± 0.07		0.52 ± 0.51	0.57 ± 0.51
	Glucose	0.948 [a]		-0.39 ± 1.31		0.00 ± 0.00	0.30 ± 0.47
		**0.948 [a]**		**-1.37 ± 0.88**			**1.00 ± 0.00**
	Potassium	0.961 [a]		0.06 ± 0.05		0.61 ± 0.50	0.65 ± 0.49
L (ml/min)		0.75 ± 2.57				0.3[a]	0.3[a]
		**0.12 ± 1.27**				**0.3[a]**	**0.3[a]**
**Weight** (kg)				80.8±38.4			
				**85±17**			

[a]Parameter fixed in the simulation.

[b]MTAC sodium was mostly negative in the simplistic models (GM, WM and SWM) due to ignoring sodium sieving.

3. Subject-specific simulations

3.1. Specific-pig simulations

3.1.1. Predicted values for 6 models in a representative pig dwell (1)

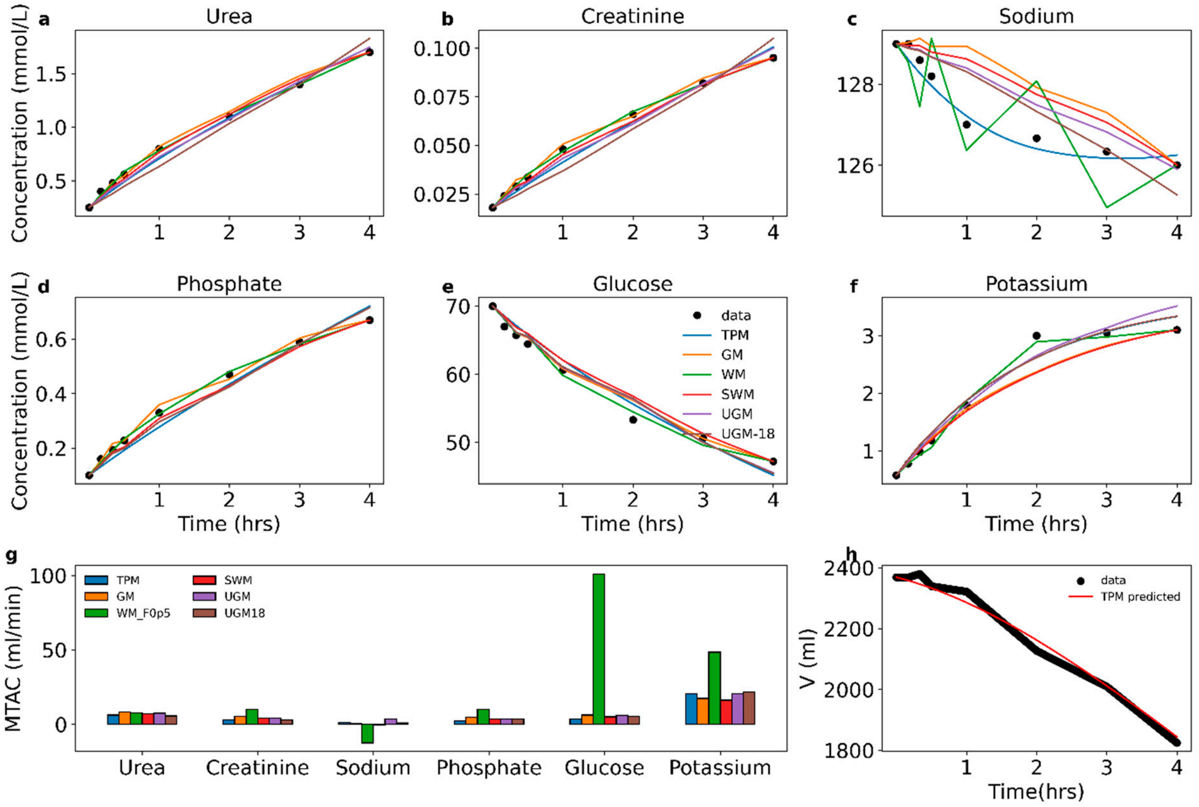

Figure S1: (a-f) Predicted dialysate concentration by each model after going through 10 iterations to find the best fit for in a representative pig dwell. (g) Predicted MTAC values for all solutes. (h) TPM predicted intraperitoneal volume profiles against measured volume (used for all other models).

3.1.2. Predicted parameter values for 6 models in representative pig dwell (2)

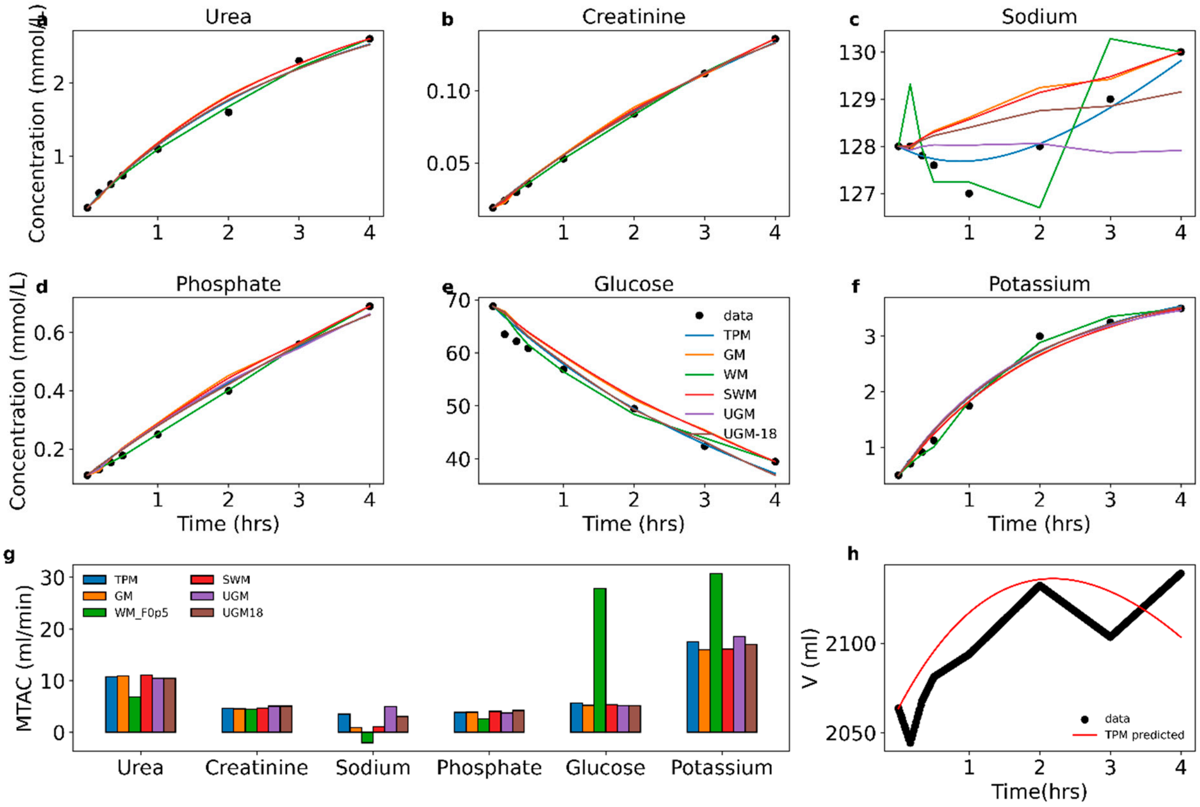

Figure S2: (a-f) Predicted dialysate concentration by each model after going through 10 iterations to find the best fit for in a representative pig dwell. (g) Predicted MTAC values for all solutes. (h) TPM predicted intraperitoneal volume profiles against measured volume (used for all other models).

3.1.3. Predicted parameter values for 6 models in representative pig session (3)

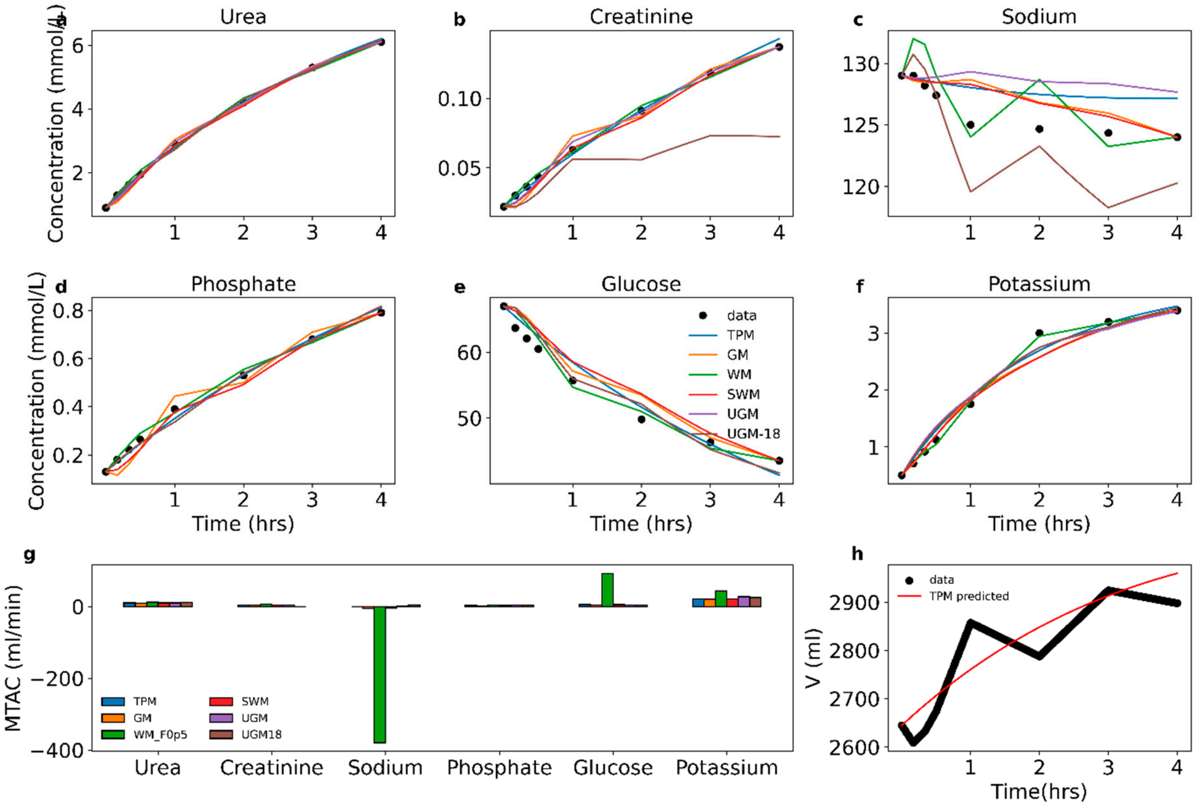

Figure S3: (a-f) Predicted dialysate concentration by each model after going through 10 iterations to find the best fit for in a representative pig dwell. (g) Predicted MTAC values for all solutes. (h) TPM predicted intraperitoneal volume profiles against measured volume (used for all other models).

3.1.4. Predicted parameter values for 6 models in representative pig dwell (4)

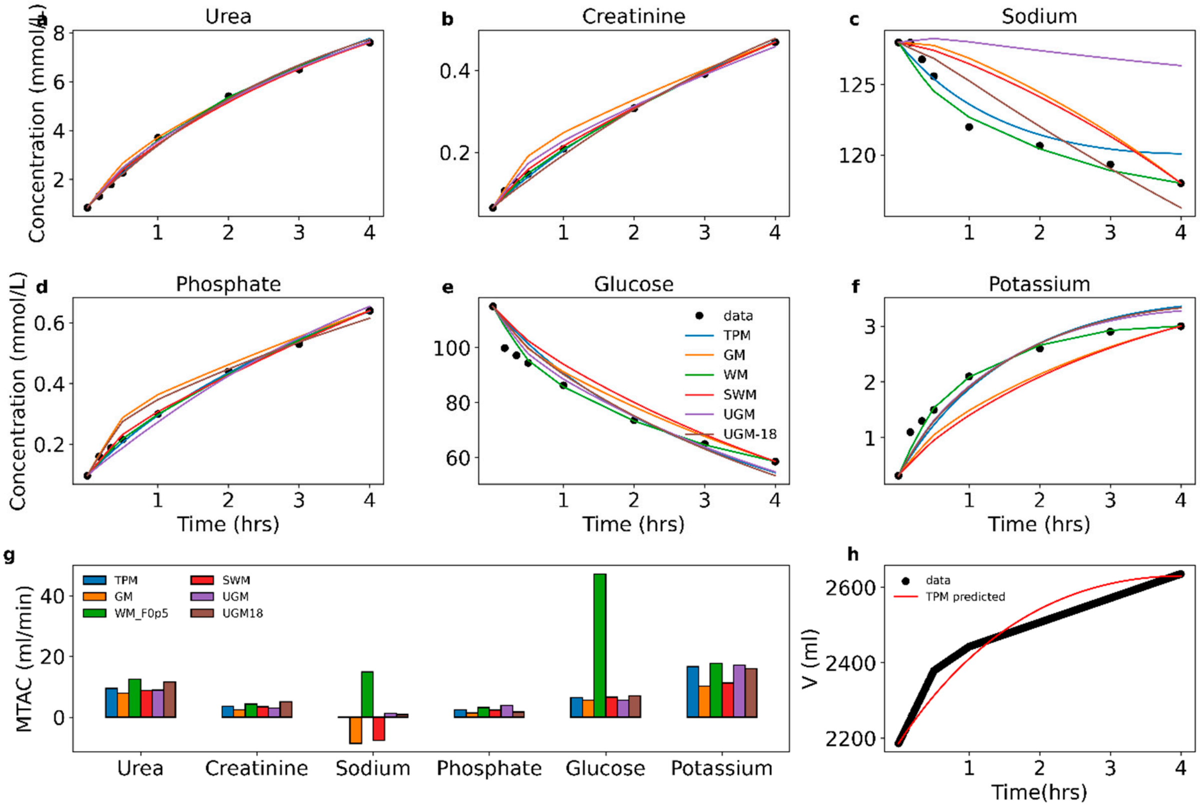

Figure S4: (a-f) Predicted dialysate concentration by each model after going through 10 iterations to find the best fit for in a representative pig dwell. (g) Predicted MTAC values for all solutes. (h) TPM predicted intraperitoneal volume profiles against measured volume (used for all other models).

3.2. Specific-human simulation

3.2.1. Predicted parameter values for 6 models in representative human dwell (1)

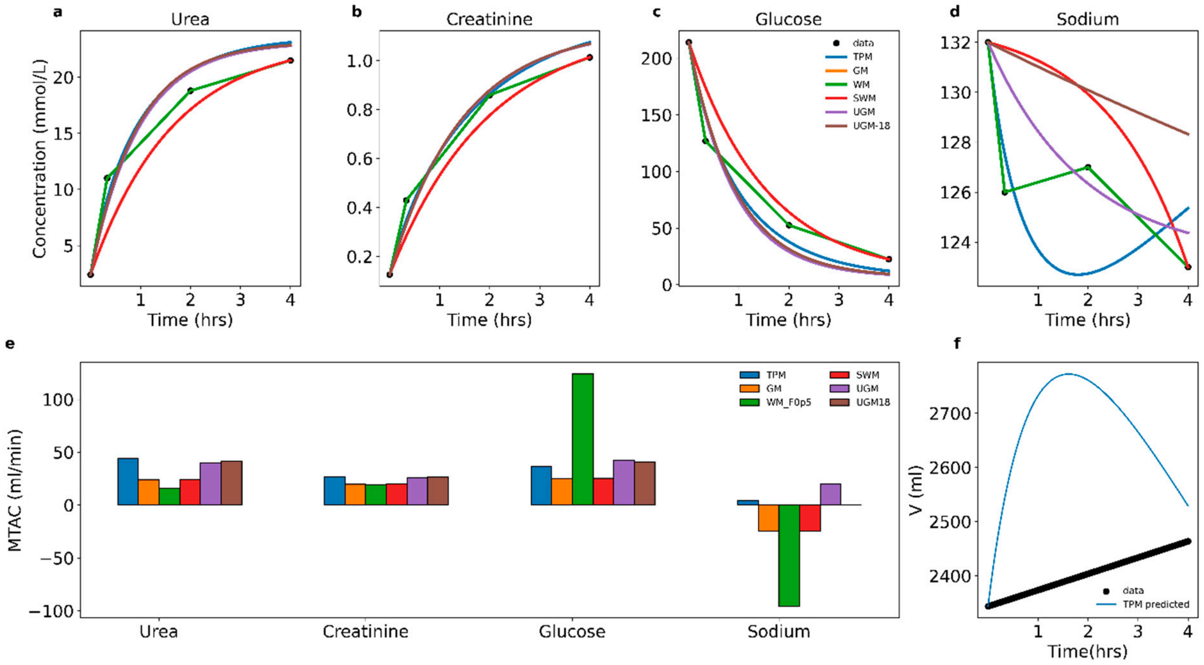

Figure S5: (a-d) Predicted dialysate concentration by each model after going through 10 iterations to find the best fit for in a representative human dwell. (e) Predicted MTAC values for all solutes. (f) TPM predicted intraperitoneal volume profiles against measured volume (used for all other models).

3.2.2. Predicted parameter values for 6 models in representative human dwell (2)

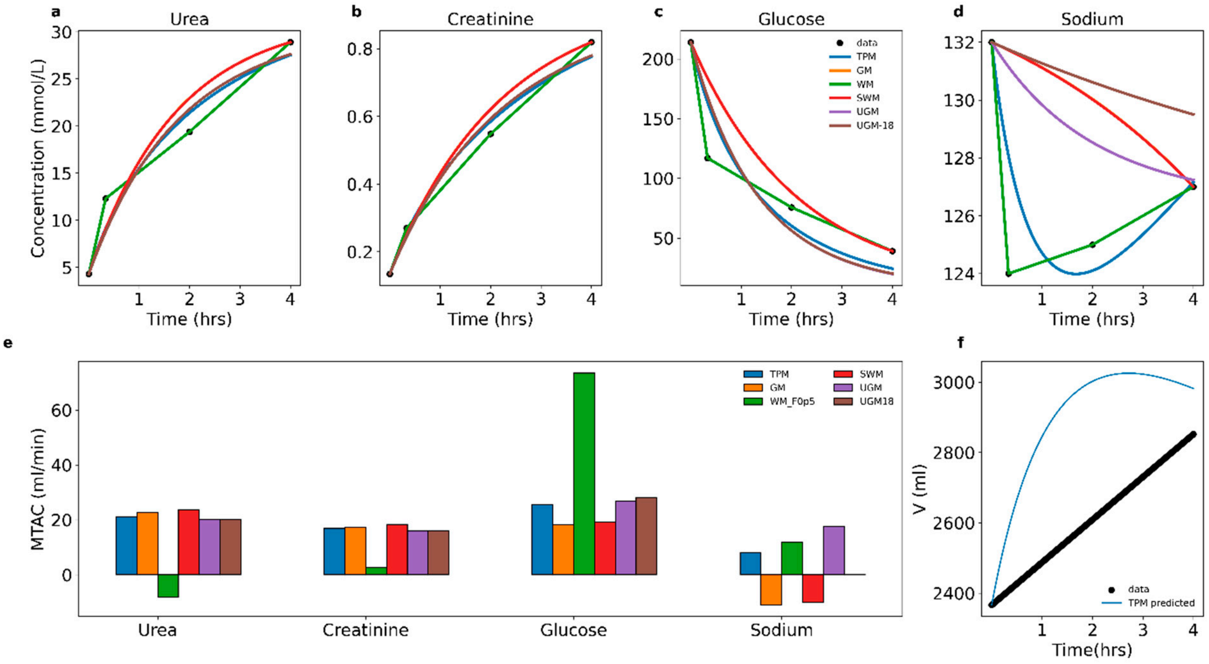

Figure S6: (a-d) Predicted dialysate concentration by each model after going through 10 iterations to find the best fit for in a representative human dwell. (e) Predicted MTAC values for all solutes. (f) TPM predicted intraperitoneal volume profiles against measured volume (used for all other models).

3.2.3. Predicted parameter values for 6 models in representative human dwell (3)

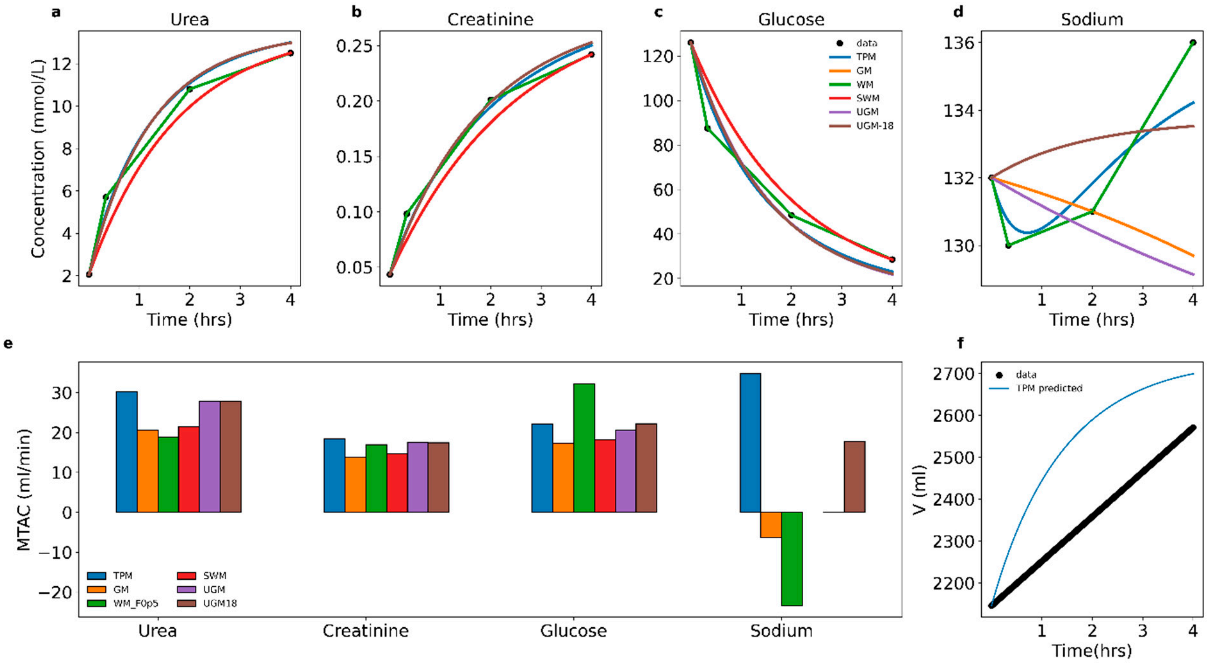

Figure S7: (a-d) Predicted dialysate concentration by each model after going through 10 iterations to find the best fit for in a representative human dwell. (e) Predicted

MTAC values for all solutes. (f) TPM predicted intraperitoneal volume profiles against measured volume (used for all other models).

3.2.4. Predicted parameter values for 6 models in representative human dwell (4)

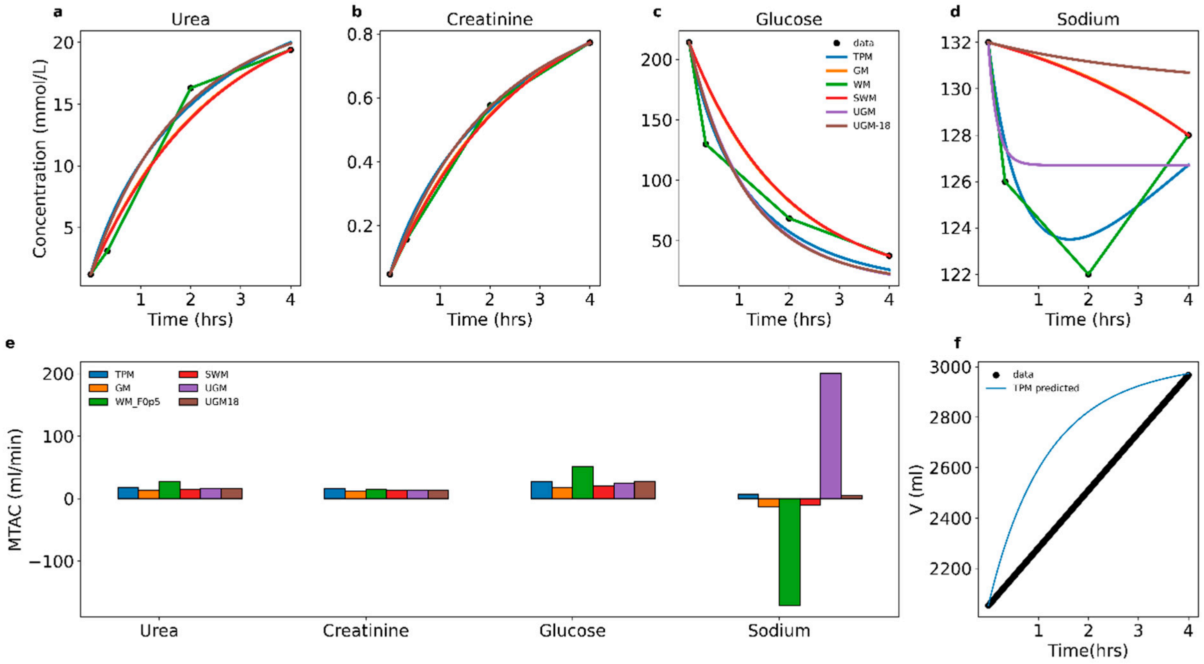

Figure S8: (a-d) Predicted dialysate concentration by each model after going through 10 iterations to find the best fit for in a representative human dwell. (e) Predicted MTAC values for all solutes. (f) TPM predicted intraperitoneal volume profiles against measured volume (used for all other models).

3.3. Population-average of fitted parameters

3.3.1. Fitted and derived fct values

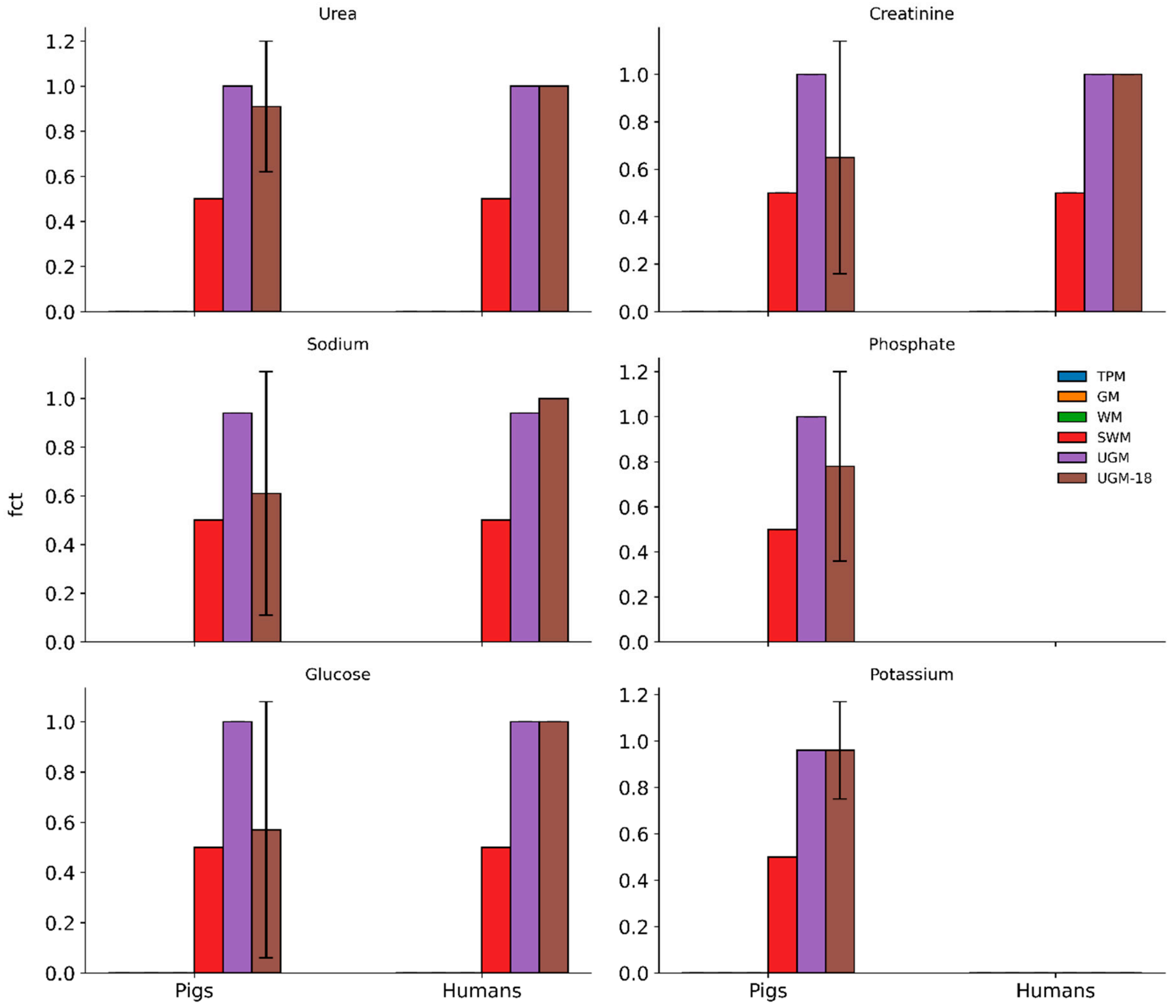

Figure S9: Model predicted mean solute fcts comparison within the models. For GM, WM and SWM, fct is fixed at 0, 0 and 0.5 respectively. UGM fct values are fixed as per **Table** S2. UGM-18 fct values for humans were all predicted to be 1. The parameter is implicitly used for TPM and not calculated separately.

3.3.2. Fitted and derived sieving coefficient values

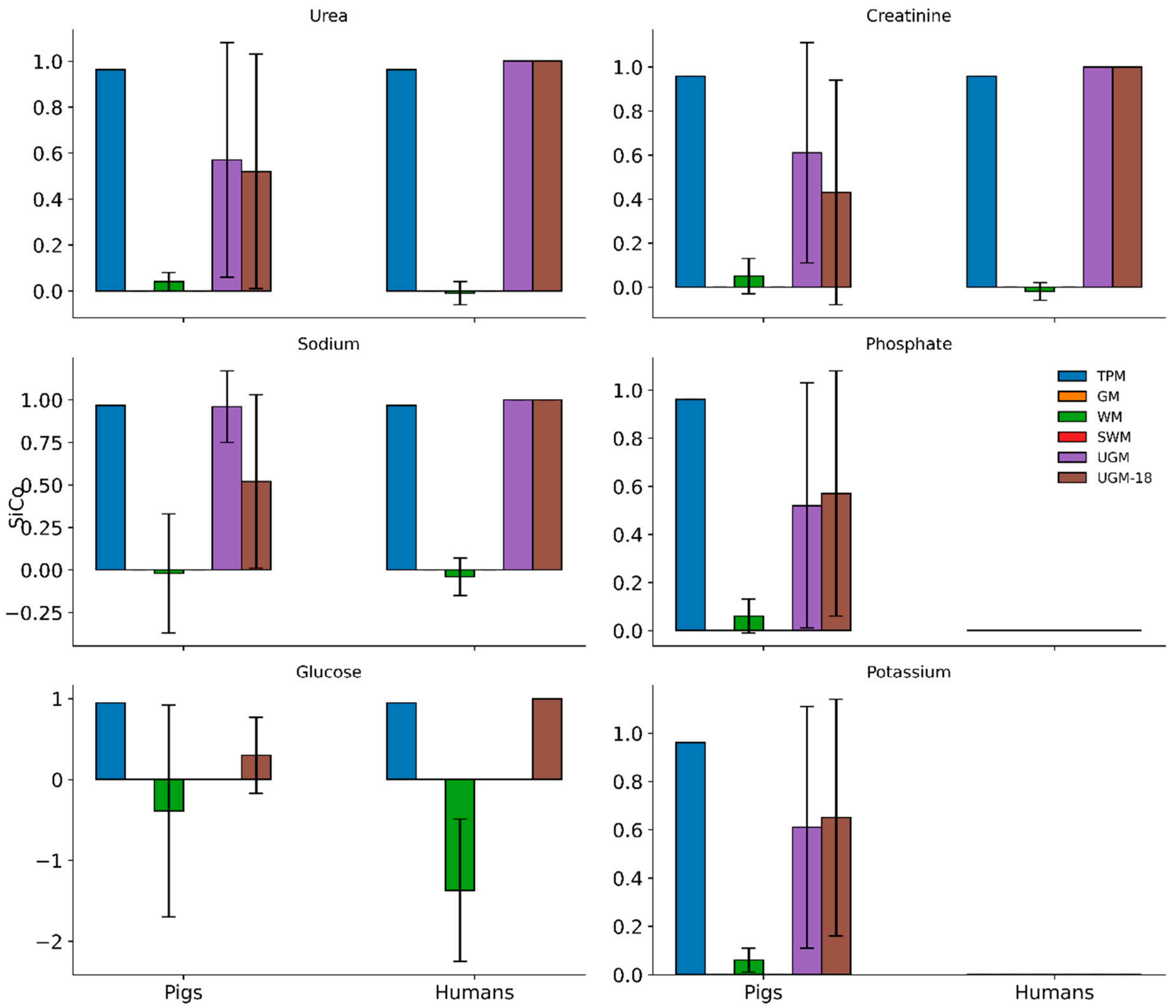

Figure S10: Model predicted mean solute sieving coefficient comparison within the models. TPM SiCo values are fixed according to **Table** S2. For UGM, SiCo for glucose is fixed at 0.

3.4. Correlation matrix

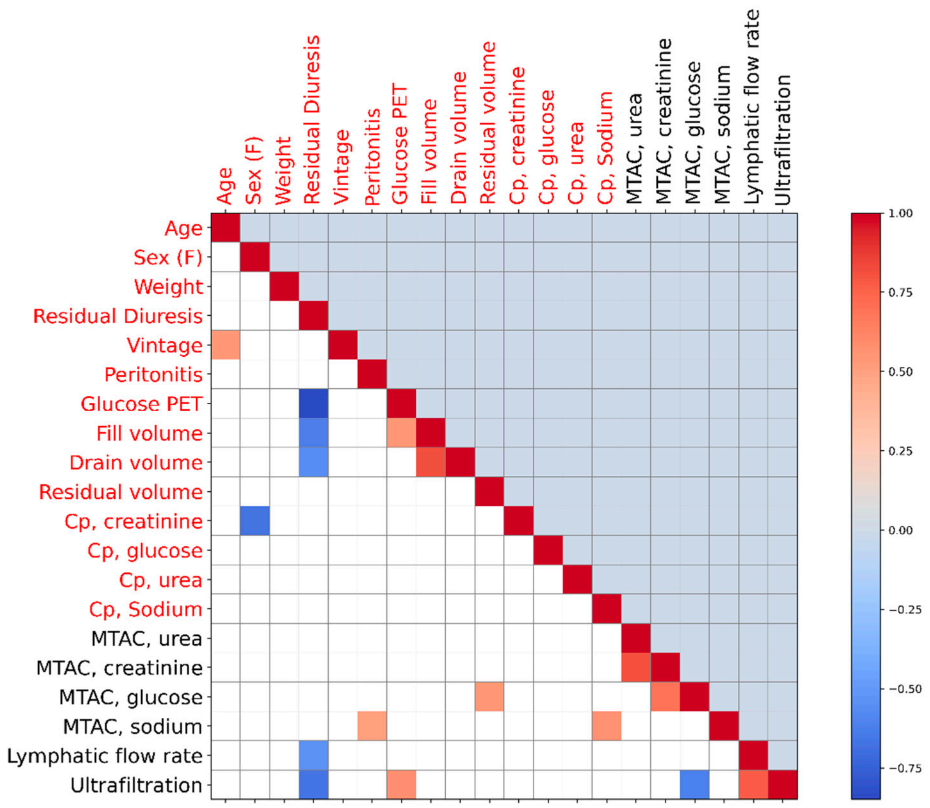

Figure S11: Correlation matrix displaying the relationships between TPM-predicted parameters (MTACs for urea, creatinine, sodium and glucose, lymphatic flow rate and total ultrafiltration) as well as other pertinent measured clinical parameters (shown in red) including age, sex, weight, residual diuresis, dialysis vintage and history of peritonitis. Higher positive (red) or negative values (blue) indicate stronger correlations between the corresponding variables, providing insights into potential dependencies and influencing factors within the dataset. Only strong dependencies are shown here to maintain clarity.

For **Figure** S11, we constructed a correlation matrix to comprehensively analyse the interrelationships between patient demographics and predicted parameters in the dataset. This analysis aims to uncover potential patterns, dependencies, and associations among these variables. Understanding these correlations provides valuable insights into how different factors might influence MTAC values and guide further investigations, support decision-making processes, and contribute to the development of more accurate predictive models or targeted interventions in peritoneal dialysis.

As expected, we see that the small solute MTACs correlate. For patient demographics we did not see a lot of strong correlations. Ultrafiltration (ml) is strongly related to

glucose PET % (0.616) and MTAC glucose (-0.607). Lymphatic flow rate (ml/min) is strongly negatively correlated to residual diuresis (-0.669) and strongly positively correlated to glucose PET % (0.846).

3.5. Avenues for personalisation of treatment for patients

For optimising PD treatment solute clearance, ultrafiltration and glucose exposure need to be considered[11]. We created a novel treatment score matrix to characterise the PD treatment based on dialysis settings (glucose concentration and dwell time) aiming for maximal weighted sum of urea clearance and ultrafiltration rate, also considering the amount of glucose absorbed (via heat mapping). We chose urea as our solute of interest here, because urea concentration is typically used to assess dialysis adequacy (although we acknowledge its limitations[12]) and urea was predicted best by all models, including TPM. Assuming calculation time could be reduced using advanced computational power, we chose TPM to optimise dwell time and glucose starting concentration for optimum treatment of a selected patient (**Figure** S12).

Purpose was to assign a treatment score for various dialysis settings (glucose dialysate concentration and dwell time). After normalising the predicted solute removal and ultrafiltration by the maximum values of each, we could compute a treatment score for clinicians to consider before selecting the dwell time and the glucose concentration. We used the TPM model to calculate the clearances and UF values. We chose three glucose concentration, namely 1.36, 2.27 and 3.86% w/v glucose (Physioneal) and three dwell session durations of 1, 2 and 4 hours.

$$Treatment\ Score = w_{Cl} * \frac{\frac{Cl_{urea}}{Cl_{urea_{max}}}}{Glu_{absorbed}} + w_{UF} * \frac{\frac{F}{UF_{max}}}{Glu_{absorbed}} \qquad 2.6$$

where w_{Cl} and w_{UF} are the weights given to clearance and ultrafiltration respectively, Cl_{urea} is urea mass removed divided by the plasma urea concentration, and UF is the total ultrafiltration calculated in ml. The weights, w_{Cl} and w_{UF} were chosen in the range of 0 to 1 such that their sum is equal to 1. The treatment score also takes into account the amount of glucose absorbed ($Glu_{absorbed}$), which should preferably be kept low, under the different settings of glucose solutions and dwell time. We used MinMaxScaler from Python package sklearn to normalise treatment score and $Glu_{absorbed}$. MinMaxScaler scales each feature to a specified range, typically [0, 1], using the formula

$$X_{scaled} = \frac{X - X_{min}}{X_{max} - X_{min}} \quad 2.7$$

Clinicians can then use the highest treatment score to select the dialysis setting for the specific patient, keeping in check the glucose absorption amounts.

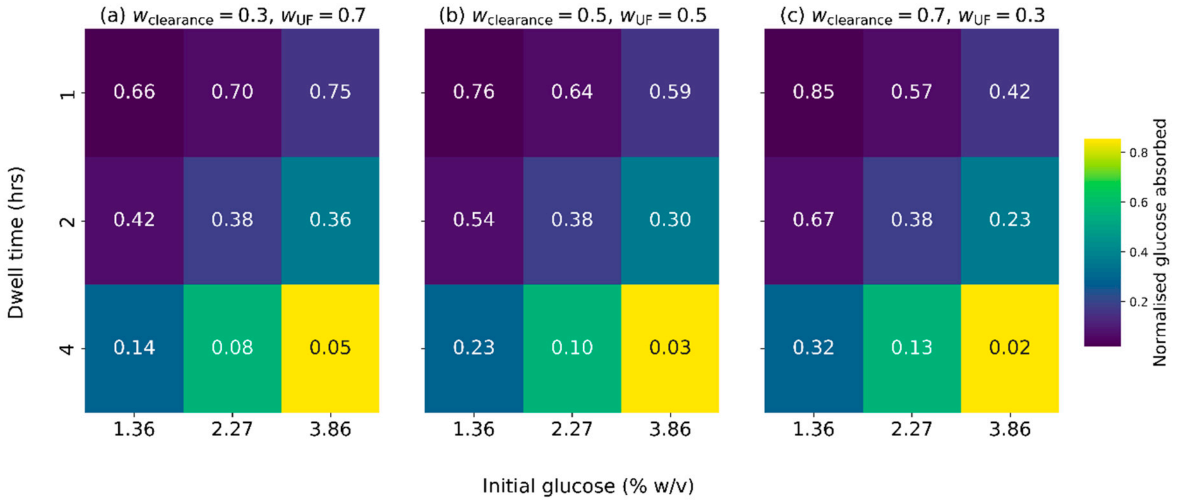

Figure S12. Treatment score matrix for three different weight distribution ($w_{clearance}$ = weight associated with urea clearance, w_{UF} = weight associated with ultrafiltration rate). (a) Higher priority on ultrafiltration. (b) Equal priority between urea clearance and ultrafiltration. (c) Higher priority on urea clearance. The heat maps are colored to represent the amount of glucose absorbed, where lighter hues represent higher amount. The higher the numerical value, better is the option, but keep in mind the amount of glucose absorbed.

References

1. Graff, J.; Fugleberg, S.; Joffe, P.; Brahm, J.; Fogh-Andersen, N. An evaluation of twelve nested models of transperitoneal transport of urea: the one-compartment assumption is valid. *Scandinavian Journal of Clinical and Laboratory Investigation* **1995**, *55*, 331-339.
2. Graff, J.; Fugleberg, S.; Joffe, P.; Fogh-Andersen, N. Parameter estimation in six numeric models of transperitoneal transport of glucose. *ASAIO Journal (American Society for Artificial Internal Organs: 1992)* **1994**, *40*, 1005-1011.
3. Graff, J.; Fugleberg, S.; Joffe, P.; Brahm, J.; Fogh-Andersen, N. Parameter estimation in six numerical models of transperitoneal transport of potassium in patients undergoing peritoneal dialysis. *Clinical Physiology* **1995**, *15*, 185-197.

4. Fugleberg, S.; Graff, J.; Joffe, P.; Løkkegaard, H.; Feldt-Rasmussen, B.; Fogh-Andersen, N.; Nielsen, S.L. Transperitoneal transport of creatinine. A comparison of kinetic models. *Clinical Physiology* **1994**, *14*, 443-457.
5. Graff, J.; Fugleberg, S.; Brahm, J.; Fogh-Andersen, N. Transperitoneal transport of sodium during hypertonic peritoneal dialysis. *Clinical Physiology* **1996**, *16*, 31-39.
6. Graff, J.; Fugleberg, S.; Brahm, J.; Fogh-Andersen, N. The transport of phosphate between the plasma and dialysate compartments in peritoneal dialysis is influenced by an electric potential difference. *Clinical Physiology* **1996**, *16*, 291-300.
7. Rippe, B. A Three-Pore Model of Peritoneal Transport. *Peritoneal Dialysis International* **1993**, *13*, 35-38, doi:10.1177/089686089301302S09.
8. Drake, R.; Davis, E. A corrected equation for the calculation of reflection coefficients. *Microvascular Research* **1978**, *15*, 259.
9. Garred, L.J.; Canaud, B.; Farrell, P.C. A simple kinetic model for assessing peritoneal mass transfer in chronic ambulatory peritoneal dialysis. *asaio J* **1983**, *6*, 131-137.
10. Waniewski, J.; Werynski, A.; Heimbürger, O.; Lindholm, B. Simple Models for Description of Small-Solute Transport in Peritoneal Dialysis. *Blood Purification* **1991**, *9*, 129-141, doi:10.1159/000170009.
11. Li, P.K.-T.; Chow, K.M.; Cho, Y.; Fan, S.; Figueiredo, A.E.; Harris, T.; Kanjanabuch, T.; Kim, Y.-L.; Madero, M.; Malyszko, J.; et al. ISPD peritonitis guideline recommendations: 2022 update on prevention and treatment. *Peritoneal Dialysis International: Journal of the International Society for Peritoneal Dialysis* **2022**, *42*, 110-153, doi:10.1177/08968608221080586.
12. Vanholder, R.; Glorieux, G.; Eloot, S. Once upon a time in dialysis: the last days of Kt/V? *Kidney International* **2015**, *88*, 460-465, doi:https://doi.org/10.1038/ki.2015.155.
